# Closing the loop: High-speed robotics with accelerated neuromorphic hardware

**DOI:** 10.3389/fnins.2024.1360122

**Published:** 2024-03-26

**Authors:** Yannik Stradmann, Johannes Schemmel

**Affiliations:** Kirchhoff-Institute for Physics, Heidelberg University, Heidelberg, Germany

**Keywords:** analog neuromorphic computing, spiking neural networks, neurorobotics, closed-loop controller, high-speed robotics, motor control

## Abstract

The BrainScaleS-2 system is an established analog neuromorphic platform with versatile applications in the diverse fields of computational neuroscience and spike-based machine learning. In this work, we extend the system with a configurable realtime event interface that enables a tight coupling of its distinct analog network core to external sensors and actuators. The 1,000-fold acceleration of the emulated nerve cells allows us to target high-speed robotic applications that require precise timing on a microsecond scale. As a showcase, we present a closed-loop setup for commuting brushless DC motors: we utilize PyTorch to train a spiking neural network emulated on the analog substrate to control an electric motor from a sensory event stream. The presented system enables research in the area of event-driven controllers for high-speed robotics, including self-supervised and biologically inspired online learning for such applications.

## 1 Introduction

Often perceived to be one of the key features of biological cognitive systems, embodiment has become subject to increasing interest within the neuroscientific community (Kiverstein and Miller, 2015; Deng et al., 2019). While state-of-the-art neural networks often outperform humans in cognitive tasks, artificial agents still severely lack behind their mammalian counterparts in applications requiring precise control and coordination of movement. The field of neurorobotics tries to overcome these deficits by tightly interconnecting physical agents to brain-inspired computing solutions. This approach promises multiple advantages compared to the classical separation of control and cognition: on the one hand, it allows research on how to leverage the advantage biological nervous systems show when executing movement in complex environments. On the other hand, our understanding for artificial cognitive systems may improve when their response to a permanent stream of complex real-world stimuli is examined. The required tight coupling can be achieved by linking simulations of biologically inspired neural networks to either real-world robots (Hagras et al., 2004) or virtual agents in simulated environments (Falotico et al., 2017). While the latter in principle allows for arbitrary complexity in both, the physics and the neural network simulation, runtime constraints often lead to significant size and fidelity limits for both components. Physical robots, in contrast, inherently experience the rich dynamics of a real-world environment. This poses strong timing requirements on the—simulated—nervous system, which needs to interact with its surroundings in realtime. Especially for biologically inspired spiking neural networks (SNNs), this prerequisite has led to the deployment of specialized neuromorphic accelerators for neurorobotic tasks (Richter et al., 2016; Blum et al., 2017; Milde et al., 2017; Kreiser et al., 2018; Yan et al., 2021; DAngelo et al., 2022). These systems integrate specific analog or digital modules for the efficient simulation—or *emulation*—of spiking neurons and are thereby capable of running such networks at biological realtime. This makes them a versatile tool for robotic applications operating on time scales similar to those accessible by their biological example.

In contrast to these established neurorobotic platforms, the accelerated BrainScaleS-2 (BSS-2) system operates with time constants that are 1,000-fold accelerated compared to their biological counterparts, allowing the system to tackle robotic tasks far beyond human speed (Schreiber, 2021). In this work, we present a flexible realtime event interface for BSS-2 and showcase its application with a simple closed-loop controller for brushless electric motors. This problem is well-studied and has been solved with a multitude of different algorithms, both for senosored and sensorless applications (Pillay and Krishnan, 1989; Ko et al., 1993; Acarnley and Watson, 2006; Rodriguez and Emadi, 2007; Sathyan et al., 2009; Bosso et al., 2021). While an applicable agent can be built with affordable off-the-shelf components, it does require precise timing far beyond human performance and—due to the ubiquity of electric motors—still has high technological relevance. To showcase the realtime event interface for BSS-2, we therefore present a basic controller for sensored brushless motors that has been trained to mirror the behavior of a classical controller using standard machine-learning techniques and hardware-in-the-loop training (Schmitt et al., 2017; Yao et al., 2020). We envision this setup to be a versatile platform for research on the interaction of biologically plausible spiking neural networks with fast physical systems.

## 2 A realtime event interface for BrainScaleS-2

**Figure 1:**
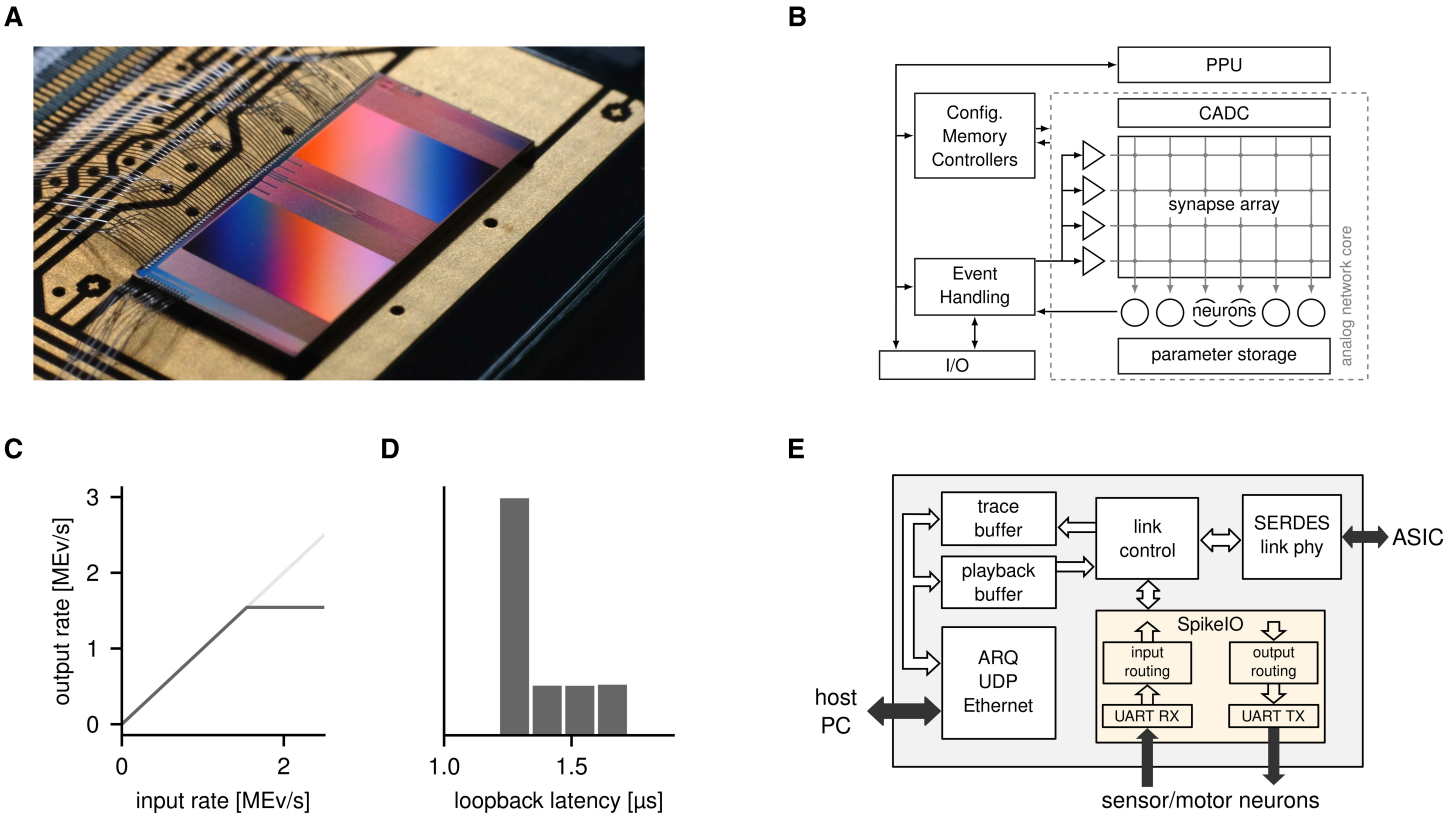
**(A)** Photograph of a BrainScaleS-2 (BSS-2) ASIC. **(B)** Schematic diagram of the BSS-2 ASIC with its distinctive analog network core. Figure adapted from Billaudelle et al. (2020). **(C)** Bandwidth measurement of the BSS-2 event interface. The presented realtime interface saturates at 1.5 MEv s-1 (full duplex), allowing for significant event traffic to/from external sensor and motor signals. **(D)** Loopback latency measurement of the presented BSS-2 event interface. We observe a mean roundtrip time of 1.2 μs for a spike to travel from the ASIC, through the realtime event interface and back into the ASIC. **(E)** Block-level diagram of the FPGA which accompanies the BSS-2 ASIC. The additions presented in this work have been highlighted in yellow: a bidirectional serial interface allows events from sensor and motor neurons to be injected/extracted in realtime. Routing tables for mapping events to/from the ASIC to virtual spike channels are configurable at runtime.

BSS-2 is a hybrid neuromorphic system that has primarily been designed to facilitate research of biologically inspired SNNs (Figures 1A, B; Pehle et al., 2022). In its core, it features 512 individually configurable analog neurons, each of which is capable of reproducing the rich dynamics of the adaptive-exponential (AdEx) neuron model with high fidelity (Brette and Gerstner, 2005; Billaudelle et al., 2022). Multiple of these neuron circuits can be combined to implement multi-compartment neurons and thereby resemble the spatial structure of biological dendrites (Kaiser et al., 2022). The neurons receive input from a total of 131,072 synapses per chip, each featuring a digitally programmable weight of 6 bit resolution. These synapses can be plastic on various different time scales: while short-term plasticity is natively integrated into the analog synapse array, spike-timing-dependent plasticity is implemented through the combination of synapse-local correlation sensors with two embedded SIMD processors. These plasticity processing units (PPUs) can be programmed to execute arbitrary C^+^^+^ code at runtime and therefore be used to realize user-defined plasticity rules on-chip. To do so, they have access to various different observables in the analog and digital domain, e.g., event counters, membrane potentials or the correlation signals mentioned previously.

Experiments on the BSS-2 system can be formulated using two different software frontends: for SNNs focussing on the complex dynamics found in biological systems, a backend for the PyNN meta-language (Davison et al., 2009) is provided. Workloads with a focus on computational neuroscience and machine learning can rely on native integration into PyTorch (Paszke et al., 2019), which especially allows for hardware-in-the loop training paradigms (Müller et al., 2022). In both cases, the experiment can be formulated in the Python programming language—the BSS-2 software stack will then compile a stream of instructions on a host computer and transfer it to the neuromorphic system.

Due to the tight realtime requirements of the system, these instructions are executed on an intermittent FPGA, which is accessible via Gigabit Ethernet. It buffers the instruction stream compiled on the host computer, streams the data to the BSS-2 ASIC, buffers network responses from the neuromorphic chip and sends them back to the host (Figure 1E, white components). As part of this work, we have extended this FPGA by a second event interface, which allows for event injection and extraction in realtime (Figure 1E, yellow components) and thereby enables robotic applications with the BSS-2 system.

To allow for a substantial amount of (virtual) spike channels without excessive I/O pin demands, we have chosen a serial protocol for receiving and transmitting sensor and motor events. Specifically, we integrate an Universal Asynchronous Receiver/Transmitter (UART), which is widely supported across all classes of embedded devices. Since the realtime nature of the application does not require time-stamped events, any word communicated over this interface represents the channel ID of an event happening at the current time. The word size—and therefore the amount of virtual event channels—can be configured at build time, while the bitrate is a runtime parameter that can be adapted to the requirements of different external spike sources and sinks. For the data link layer we rely on an open-source implementation,^1^ which uses octuple oversampling to decode events received via the asynchronous interface. With a system clock of 125 MHz and a single start and stop bit, we therefore expect a serialization latency of


(1) $$\begin{array}{l} t_{\text{serial}}={\left(\frac{125\mathrm{MHz}}{8}\right)}^{-1}\cdot \left(w+2\mathrm{bit}\right)=640\mathrm{ns}, \end{array}$$

where we have set an exemplary interface width of *w* = *8bit* (256 event channels). Correspondingly, the link layer is expected to saturate at $B_{\text{max}}=t_{\text{serial}}^{-1}=1.56MEvs\text{-1}$. The measurements depicted in Figures 1C, D indicate that these values are reached in the physical implementation: while the measured saturated event rate of *1.5MEvs*-1 directly matches the expected *B*_max_, the measured median loopback latency of $\tilde{t_{\text{lb}}}=1.2\mu s$ results from the sum of *t*_serial_ and the serialization latency between FPGA and ASIC. The latter is expected to add an additional delay of at least 268 ns in each direction.

For translation between external events and the BSS-2 spike format, individual routing tables for the sending and receiving directions are connected to the UART module. These tables utilize block RAM on the FPGA to provide a dense, runtime-configurable mapping between external event addresses and those used by the neuromorphic system. Users can query the BSS-2 software for the spike labels assigned to their sensor and motor neuron populations. These values can then be used to program an arbitrary mapping to external event channel IDs.

## 3 Showcase: BLDC motor control

To showcase the application of the presented realtime event interface in a real-world scenario, we have selected the task of commuting an electric brushless DC (BLDC) motor. We have chosen this problem due to the simplicity of the physical setup and its precise timing requirements: BLDC motors—together with sensor and power electronics—are widely available off-the-shelf components, yet they rely on precisely controlled currents to operate reliably. With typical rotational speeds far above 10,000 min^–1^, the timing requirements for enabling individual coils are in the sub-millisecond regime and therefore go far beyond human capabilities (Kemp, 1973). At the same time, the task is particularly suitable for biologically-inspired spiking neural networks (SNNs), since the bipolar interaction of a permanent magnet with the current-induced electromagnetic field of the coils matches the concept of antagonistic muscle pairs in vertebrates.

**Figure 2:**
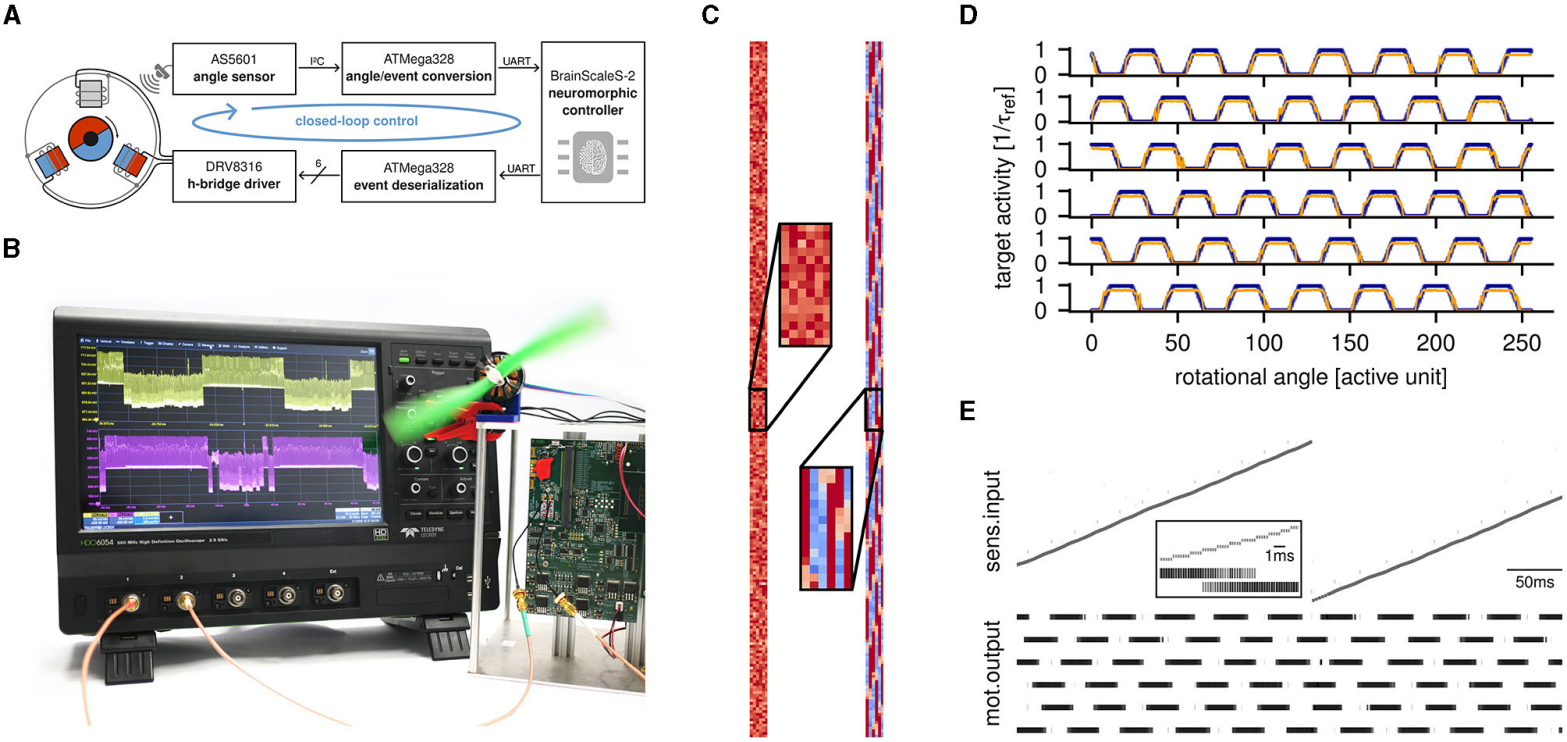
**(A)** Block diagram of the implemented closed-loop motor controller. The rotational angle is measured, converted to events and sent to the BSS-2 system in realtime. These sensory inputs are then processed by the spiking motor controller. Its motor outputs are converted to control signals for an H-bridge driver, which controls the coils of a BLDC motor. **(B)** Experimental setup for running the closed-loop experiment. The neuromorphic system is depicted on the right, with the BSS-2 ASIC hidden below the red dust cap. It controlls the small electric motor at the top, which drives a green propeller. The oscilloscope displays the membrane potential of two analog motor neurons on BSS-2. **(C)** Network weights before (left) and after (right) supervised training. The mapping between sensory input (vertical dimension) to motor output (horizontal dimension) is clearly visible after training. **(D)** Mapping from rotational angle to motor output. The blue data depicts a measurement of the behavior shown by a classical controller for BLDC motors. The orange curves represent spike rates measured on BSS-2 with simulated input after training. They have been normalized to 1/τ_ref_. **(E)** Rasterplot of the spiking motor controller during the closed-loop experiment. The motor neurons directly map to the six controllables of an H-bridge, while the sensory neurons represent the rotational angle of the motor. The transition phase for a single state shift is highlighted through magnification.

For the particular demonstration, we use a small gimbal motor with 12N14P configuration and a nominal rotation rate of 205 min-1. Its current rotational angle is read out with 10 bit resolution by a magnetic sensor, which communicates these values via I^2^C to a microcontroller (Figure 2A, top). The latter serves as a bridge between the sensor and the BSS-2 event interface and is primarily used for encoding the current rotational angle into spikes. Algorithmically, we choose a value-unit representation, for which we pool the 1,024 available raw states into 256 sensor neurons. The resulting events are then injected into the analog network emulation on BSS-2 and processed by a single feed-forward layer of six leaky integrate-and-fire (LIF) neurons. Taking inspiration from biology, we utilize pulse-density modulation for the efferent motor signals. Similar to aforementioned antagonistic muscle pairs in biology, the activity of the output neurons $M_{j}^{i}$ directly corresponds to the state of the six transistors controlling the motor: each of the three phases *i* can either be driven high (only $M_{\text{high}}^{i}$ active), driven low (only $M_{\text{low}}^{i}$ active), or left floating. The decoding of the output event stream is handled by a second microcontroller, which momentarily enables the corresponding signal in a 6 bit parallel bus whenever a motor neuron spikes (Figure 2A, bottom). These signals are used by the output stage of a commercial motor driver to control an internal 3-phase H-bridge. Any activity of the motor neurons emulated on BSS-2 thereby creates physical movement, which in turn results in a change of the rotational angle and therefore potentially different inputs to the SNN.

We trained this SNN-based motor controller using standard machine-learning techniques in PyTorch. To do so, we have recorded the behavior of an open-source reference BLDC motor controller (Skuric et al., 2022), lowpass-filtered the PWM outputs, and created a mapping of the current rotational angle to the resulting target activity per motor neuron $M_{j}^{i}$ (Figure 2D, blue curves). With this dataset at hand, we were able to train the SNN emulated on BSS-2 with the established hardware-in-the-loop paradigm: during training, the forward pass is evaluated on the analog neuromorphic substrate, while the gradients are computed on an idealized, differentiable model for the neuron's behavior. The presented results have been trained for 5 epochs against a L2 loss function with the Adam optimizer (Kingma and Ba, 2014), all parameters are summarized in Table 1. The orange curves in Figure 2D show the response of the hardware neurons for equal test stimuli across 20 trials post training. The strength of the neuronal response has been normalized to the inverse of the neuron's refractory time τ_ref_, as this poses an—unreachable—upper limit for the firing rate of every cell. Figure 2C shows the weight matrix of the feed-forward layer before (left) and after (right) training: when training from an initially random weight matrix, the correct mapping between the current angular position (vertical axis) and the corresponding active motor neuron (horizontal axis) clearly emerges.

**Table 1:** Parameters for the presented SNN-based BLDC controller.

Neuron model	Leaky integrate-and-fire
Membrane time constant τ_mem_	20 μs
Refractory time τ_refr_	32 μs
Synaptic time constant τ_syn_	20 μs
Network architecture	Single layer feed-forward SNN, 6 units
Sensory input coding	Value-unit, rate-based
Motoric output coding	Value-unit, pulse-density modulated
Training	Stochastic gradient decent
Loss function	L2
Optimizer	Adam
Learning rate	0.1
β	0.9, 0.999
Weight decay	0
Weight initialization	0.5 ± 0.1

All time constants are given in wall-clock time, illustrating the accelerated nature of the analog substrate.

The full system in operation is depicted in Figure 2B, where the BSS-2 system (right side, with the ASIC hidden under a red dust cap) controls a small BLDC motor. The oscilloscope shows membrane recordings of the two motor neurons $M_{\text{low}}^{0}$ and $M_{\text{high}}^{0}$, the areas of high activity are clearly separated from those with strong inhibition. Figure 2E shows a corresponding rasterplot with all involved signals. The linearly ascending sensory inputs indicate the continuous rotational motion of the motor, while the motor neurons show the expected repetitive six-phase commutation pattern.

## 4 Discussion

This work introduced a realtime spike interface for the accelerated neuromorphic BSS-2 platform, paving the way for high-speed robotic applications. Honoring the system's fast dynamics, it has been optimized for low latency, moderate bandwidth and high compatibility with embedded components for sensor inputs and motor outputs. Similar to Romero Bermudez et al. (2023), we have aimed for a generic interface, which we envision to be suitable for a cornucopia of robotic experiments. Its low latency allows users to tackle tasks requiring timing precision on microsecond-level.

As an initial application, we have shown an SNN-based controller for BLDC motors and thereby join the rank of neuromorphic control units for motor tasks that reaches back multiple decades (DeWeerth et al., 1991). The particular application has been made possible by the 1,000-fold acceleration factor of the BSS-2 system, which enables research on the unexplored grounds of biologically inspired controllers for high-speed robotic tasks. While the presented application relies on supervised training to mimic a classical controller, the setup allows for the exploration of self-supervised and—in combination with the on-chip plasticity processors—online learning. In these cases, the acceleration factor will not only enable the application of SNNs to robotic tasks with superhuman timing requirements, but also greatly reduce their training time. For the particular example of BLDC commutation, more complex networks would benefit from additional controllables and input quantities: in the afferent path, a rich sensory event stream encoding system properties like temperatures, coil currents and voltages is possible. For the motor neurons, the implemented biologically inspired pulse-density modulation might benefit from additional efferent signals for a more refined control of the coil currents. Since we implement both, the encoding and decoding between spikes and physical quantities in microcontrollers, such alterations to the system are possible without physical access. Together with the native integration of the presented realtime event interface into the BSS-2 software stack, this allows for the remote development of high-speed robotic applications through the EBRAINS platform.^2^

## Data availability statement

The original contributions presented in the study are included in the article/supplementary material, further inquiries can be directed to the corresponding author.

## Author contributions

YS: Conceptualization, Data curation, Formal analysis, Investigation, Methodology, Project administration, Software, Validation, Visualization, Writing – original draft, Writing – review & editing. JS: Conceptualization, Funding acquisition, Methodology, Project administration, Resources, Supervision, Writing – original draft, Writing – review & editing.

## Footnotes

^1^Alex Forencich: Verilog UART, https://github.com/alexforencich/verilog-uart.

^2^ https://ebrains.eu/nmc
